# Out-Patients at St. Thomas's

**Published:** 1889-08-03

**Authors:** 


### The Hospital Workshop.

### VII.—OUT-PATIENTS AT ST. THOMAS'S.

(BY OUR SPECIAL COMMISSIONER.)

The palatial-looking building on the Surrey side of the Thames, opposite the Houses of Parliament, is probably a familiar landmark with most of our readers. St. Thomas's was founded in the year 1540. At present it stands third on the list of general hospitals as regards the number of out-patients, which amounted to 87,601 in 1887. The pay system, which is admitted in the wards of this institution, does not apply to the out-patient department, where everything is free. Applicants are sorted out, so to speak, the slight cases being attended to at once, while others, requiring more prolonged care, are furnished with a letter of admission, on which are entered name, age, occupation, and other details of the case and treatment.

The main waiting-hall is well seated and comfortable. Down one side are ranged the consulting-rooms, and on the other are no less than ten large arched windows. A passage leads to the dispensary, where, at four o'clock on the afternoon of your commissioner's visit, seven persons were standing at the wickets, and dispensing seemed to be going on at a rapid rate. Close by is a refreshment stall. Patients attend at 1.30 p.m., and at the hour previously mentioned about 200 people still remained to be seen—a fact which will give some idea of the rush on this department. On Monday and Tuesday the pressure is greatest, and Mr. G. H. Makins, assistant surgeon to the hospital, holds in the casualty wing a kind of overflow meeting, which it is the purpose of the present paper to describe briefly.

Upon entering, the surgeon was walking between the rows of applicants and distributing cards for a single visit or for regular attendance, as demanded by the nature of the case. Some of the patients are sent on to the special departments, such as the skin or throat and ear. Crossing to the women's room, the same process was gone through; and returning thence to the male side, the old cases were disposed of.

Many of the patients are of a town-bred type, stunted and unhealthy. One man in particular, with knee-joint mischief of old standing, had a face of that indescribable yellowish tint that the old painters were fond of giving to their saints and martyrs. At the same time there are plenty of healthy-looking and well-made people who have come here chiefly on account of accidents or trifling sickness. Almost the first thing that strikes one on inspecting this class of practice is the large proportion of ulcers of the leg. This is explained by the conditions of circulation, which is weakened in that part by its distance from the heart. Many of the patients lead hard laborious lives, and have to stand a great deal, which adds to the existing stress on the vessels, caused by the downward pressure of the column of venous blood. The tightly-gartered knee is also answerable for much of the damage. From all these causes an immense strain is thrown on the valves of the veins, which give way or become "varicose." Most people know the swollen and knotted appearance of that condition, and also the purple look of the skin that tells of loaded blood-vessels. Under these circumstances the limb is ready to ulcerate on very slight injury. When an ulcer is once started, absolute rest with the limb raised is the only sound treatment; but poor people cannot afford to give up their work, and the results are often disastrous. One man, for instance, shows a terrible and hopeless ulcer, a hand's breadth across, reaching round the whole leg above the ankle. In these extreme cases amputation is sometimes needed. Antiseptics have made some improvement in treatment. At St. Thomas's the ulcer is washed with a weak solution of corrosive sublimate and covered in with a gauze bandage and Unna's paste, composed of oxide of zinc and glycerine. This dressing has the advantage of being fixed and economical, and can, moreover, be kept on for a week together, which is a distinct gain to everyone concerned. Too frequent dressing or an unhealthy state of the ulcer prevent the tender scales of the skin from bridging over the wound. Healing is shown by a bluish line that creeps in from the edges. Tiny pieces of skin are often grafted on the raw surface, where, if successful, they form fresh centres of growth. A favourite plan of treatment at many hospitals is to cover in the limb with broad strips of plaster.

Here is a patient who has been in the surgeon's hands for more than two years, a good part of that time having been spent in the wards. He is a carriage-maker by trade, and his illness followed an injury to the backbone, caused by falling under the body of a coach he was helping to lift. The bones of the spine became diseased, and later on a large abscess set up inflammation of the cavity of the abdomen, or "peritonitis," as it is called. He is getting better now, but, as may be imagined, shows pretty severe traces of all his sufferings. At one time he wore a "Sayre's jacket," which, by the way, he pronounces "serge." It is an appliance named after the famous American surgeon who first introduced it, and is made by winding round and round the body plaster of Paris bandages, which set into a kind of stony case, and afford complete rest to the diseased bones. One is pleased to be able to state that this patient is in a benefit club, and he adds that most of his craft belong to some society of the kind. It appears that during health he subscribed 11s. 6d. a quarter, and for a long time after his accident drew sick pay at the rate of £1 weekly, and is now drawing half that sum.

Now and then someone asks for a certificate stating he is unable to follow his employment. This is granted by the surgeon on a form, across which is printed in red letters, "Not to be used for begging purposes."

Another patient is an asthmatic undertaker in a kind of undress, consisting of a tweed coat, black trousers, and a band round his brown felt hat. Some time ago, when getting into a trap the horse started, and he was thrown to the ground with his arm doubled up under him. One of the bones was broken just above the wrist, and he has come to see if anything can be done to remedy the stiffness which has resulted. He complains hoarsely that it interferes with his business, and he can hardly get a coffin up and down stairs. A fracture of this kind often leaves a very stiff joint, and, all things considered, he has a fair amount of movement in the wrist and hand. He is recommended to rub it well and to get some friend to work it to and fro thoroughly every day.

A father brings a small boy "to have something taken out of his mouth." Last week the mother came on the same errand, but fainted and had to go away. Judging from his pale face, the father does not seem to be in much better plight.

Here is an engineer from Isleworth with some slight skin affection, which will soon cure itself. Asked by Mr. Makins why he does not go to his own doctor, he answers plumply enough, "Oh, I don't reckon him any good!" a reply which ends the conversation on that point.

There are several hip cases, one of which is sent on for admission into the wards.

This little boy with the smart red bow has come by himself, and rattles off his tale at a great rate. He tells us he will be eleven on the 5th of July. "Father sent me here because he used to notice when he was at home that I walked sideways, and so on Sunday night he told me to get my shirt off and saw my back, and gave me a good exercise, and asked me several ways I thought it came ; and then he says, 'You had better go round to the hospital on Tuesday morning.'" The boys says his shoulder does not hurt him. On examination he is woefully thin, and his shoulder-blades certainly do stick out to an alarming extent, though there is nothing much really amiss. He says his father is a waiter at one of the Shaftesbury-avenue hotels, and comes home at two in the morning. The lad is ordered tonics and cod-liver oil.

Space will not permit more than a glance at the women's side. There are a great many children. Here is one complaining of pain in the hip. The mother is dressed in black, and gives a history that does not bode well for her little girl. She has two other children ; a third lived five years only, and her husband died of consumption. So far as she knows, there has been no injury to the hip.

They are terribly common, these cases of hip-joint disease. The mother of the next little patient is told to get a "Thomas'" splint, which is largely used nowadays, for fixing the joint. Not being able to pay for one, she is directed to get a note from the clergyman of her parish if he has a Hospital Sunday collection, and the fund will provide the necessary apparatus. Meanwhile the child is to get as much fresh air as possible in a perambulator, but must not be allowed to walk about.

And so the ball is kept rolling. Let the reader reflect that the few patients mentioned are merely a small portion of the overflow surgical division, and that this kind of thing goes on day after day throughout the year. Then he will form some idea of the strain upon members of the staff, nurses, dispensers, and attendants. Deducting Sundays, and taking the number of out-patients at 87,600 (in 1887), the daily average is 280 patients. As many of them, however, have long attendances, the actual number is probably nearer 500, and much more on certain days. An excellent rule directs that not more than twenty-five new cases are to be seen by one medical officer. In this way the surgeon or physician is more likely to do justice to his patients, to himself, and to the students. For some reason or other, one comes away strongly impressed with the vastness of the work carried on at St. Thomas's, possibly on account of the magnificent building and its abundance of room. Although, as a matter of fact, more patients are seen both at St. Bartholomew's and at the "London," but in each of those instances the accommodation is old-fashioned, and somewhat cramped. St. Thomas's is a palace among the hospitals.

